# Editorial: Women in science - gastroenterology 2023

**DOI:** 10.3389/fmed.2025.1556685

**Published:** 2025-02-24

**Authors:** Stefania Chetcuti Zammit, Carmen Peralta Uroz

**Affiliations:** ^1^Gastroenterology Department, Mater Dei Hospital, Msida, Malta; ^2^Institut d'Investigacions Biomèdiques August Pi i Sunyer, Barcelona, Spain

**Keywords:** nicotinamide phosphoribosyltransferase, PEG, colon cancer, sedation, intussusception

This editorial highlights research published in *Women in Science—Gastroenterology 2023*. There articles feature high quality research, conducted by women, related to several areas in gastroenterology namely inflammatory bowel disease, surgery related to colorectal cancer and endoscopic procedures.

Women who decide to pursue a scientific career, focusing on gastroenterology, face many challenges, including funding of research, the time constraints to juggle a clinical and research career and the process of research itself including obtaining data protection clearance, data collection and statistical analysis of data and the publication process that can be lengthy. Despite these hurdles, researchers must ensure high quality research.

There has been extensive research in the development of non-invasive markers in patients with inflammatory bowel disease that reflect disease activity. These may prove more cost effective and less invasive than endoscopic procedures to monitor disease activity following adjustment of treatment. In a study by Colombo et al., nicotinamide phosphoribosyltransferase (NAMPT) and nicotinic acid phosphoribosyl transferase (NAPRT) levels in the serum and feces of patients with IBD correlated with disease severity and response to treatment. Combining the use of non-invasive markers may increase the sensitivity of these biomarkers and allow us to achieve better disease control.

Patients with colorectal cancer have a higher risk of developing a second colorectal cancer (1). Li et al. studied the outcome of type of surgical resection for the second primary colorectal cancer in patients with a previous colorectal cancerClick or tap here to enter text.. Limited resection was associated with a better prognosis and improved overall survival than radical resection due to less post-operative non cancer problems.

Gravina et al. have demonstrated the overall safety of sedation in patients with Parkinson's disease undergoing percutaneous endoscopic transgastric jejunostomy proceduresClick or tap here to enter text.. Patients were administered atropine, midazolam, a bolus of propofol at induction followed by continuous infusion of propofol. Sedation with midazolam and propofol of patients during complex endoscopic procedures is increasingly preferred due to the overall safety and better tolerance.

Zong et al. present a case of intussusception of an enterogenous cyst (ileocaecal) presenting with anal prolapse. The 19-year-old patient underwent successful ileocaecal resection. Intussusception can occur secondary to several conditions including malignancies and coeliac disease. Anal prolapse secondary to an ileocaecal mass is a rare cause of such manifestation. In the case of masses or polyps, management involves resection or removal but other conditions such as coeliac disease causing intussusception can be managed conservatively.

Here we celebrate the effort, dedication, and work of these authors who have successfully developed quality studies whilst dealing with the pressures at their workplaces. We would like their achievements and professionalism to serve as an example for younger female gastroenterology researchers.
